# Fasting Blood Glucose and Lipid Profile Alterations following Twelve-Month Androgen Deprivation Therapy in Men with Prostate Cancer

**DOI:** 10.1100/2012/696329

**Published:** 2012-10-17

**Authors:** Hasan S. Sağlam, Osman Köse, Şükrü Kumsar, Salih Budak, Öztuğ Adsan

**Affiliations:** Urology Department, Medical Faculty, Sakarya University, Sakarya, Turkey

## Abstract

*Purpose.* In this retrospective study, we aimed to investigate the effects of androgen deprivation therapy (ADT) on blood glucose and blood cholesterol levels over a 12-month period. *Materials and Methods.* Between January 2010 and June 2012, the data of 44 patients with prostate cancer who were receiving ADT were collected from a hospital database. Patients with additional malignancy or diabetes and those who had been prescribed and were currently taking cholesterol-lowering medication were excluded from the study. Data (including fasting blood glucose levels and a cholesterol profile) were collected and analysed statistically. A *P* value <0.05 was considered statistically significant. *Results.* Twelve months after the initiation of ADT, fasting blood glucose (FBG), total cholesterol (TC), low-density lipoprotein (LDL) cholesterol, high-density lipoprotein (HDL) cholesterol, and triglyceride (TG) levels changed. FBG, TC, LDL cholesterol, and TG increased significantly (*P* = 0.009, 0.000, 0.000, and 0.000, resp.), while HDL cholesterol decreased (*P* = 0.000). *Conclusion.* ADT may increase FBG, TC, LDL cholesterol, and TG but decrease HDL cholesterol by the end of a year of treatment. Therefore, close followup may be needed as a consequence of one-year ADT regarding metabolic alterations.

## 1. Introduction

Today, androgen deprivation therapy (ADT) using gonadotropin-releasing hormone (GnRH) agonists is widely used for prostate cancer patients with metastatic, locally invasive, or high-risk localised disease [1, 2]. In these groups, ADT has been shown to improve survival. At the same time, the long-term use of ADT has been associated with a variety of pivotal side effects, including diabetes mellitus, hyperinsulinaemia, lipid metabolism disturbances, cardiovascular diseases, anaemia, and osteoporosis [3, 4]. In addition, studies in males have revealed that low testosterone levels are associated with disturbances also seen in metabolic syndrome, such as lower high-density lipoprotein (HDL) cholesterol, higher triglyceride (TG) concentrations, and increased abdominal adiposity [5–10].

Investigations on the effects of ADT have revealed conflicting results. In 1995, a study conducted on 50 patients with benign prostatic hyperplasia (BPH) showed that ADT caused an increase in total cholesterol (TC), HDL cholesterol, and TG, but the levels of LDL cholesterol remained unchanged [11]. Conversely, a recent study showed a decrease in HDL cholesterol and an increase in LDL cholesterol, TG, and TC after 12-month use of ADT in 99 men with prostate cancer [12].

We aimed to investigate the effects of ADT on blood glucose and blood cholesterol levels over a 12-month period in this retrospective study.

## 2. Materials and Methods

Data regarding 66 patients with prostate cancer who had received ADT, a treatment comprising GnRH + antiandrogen (AA) or orchiectomy + AA, were collected from the database of our hospital, a tertiary care facility, between January 2010 and June 2012. Seven patients were excluded from the study due to diabetes mellitus (DM), and four patients were not included because of their use of cholesterol-lowering medications. Over the course of the study, three participants died, and one patient moved to another city and was lost to followup. The remaining 51 patients' records regarding routine biochemical tests, hormone profiles, cancer management, lipid profiles, and fasting blood glucose (FBG) levels were collected. Seven additional patients were excluded because they had missing data. Finally, we recorded and statistically analysed the data of 44 patients before treatment and at 3 months, 6 months, and 12 months after the treatment's initiation in respect to lipid profiles and FBG levels. Of these 44 patients, 18 received an AA (bicalutamide 50 mg, 1 × 1) + GnRH (leuprolide 11.25, *n* = 9; leuprolide 22.5, *n* = 7; goserelin 10.8, *n* = 2) every three months, and the remaining 26 patients underwent bilateral orchiectomy + AA administration. The indications for receiving ADT were as follows: 19 patients had metastatic prostate cancer, 8 patients had comorbidities (cardiac failure, *n* = 3; chronic obstructive pulmonary disease, *n* = 4; chronic renal insufficiency, *n* = 1) that put them at high risk, and the others were older according to life expectancy. Five of the older patients had locally advanced disease. The patients were also divided into two groups—GnRH + AA group and orchiectomy + AA group—and 12-month outcomes were compared.

A paired *t*-test was used for the repeated measures showing normal distribution to compare pre- and posttreatment values. The Wilcoxon sum of rank test was used for the measures that did not distribute normally. To compare one-year results between the groups, an ANOVA test was used. The Mann-Whitney *U* test was applied for one-year comparison of the TG measures that were not normally distributed. The results were evaluated using the Statistical Package for the Social Sciences (SPSS) version 20.0 (SPSS Inc., Chicago, IL, USA). A *P* value <0.5 was considered statistically significant.

## 3. Results

**Figure 1:**
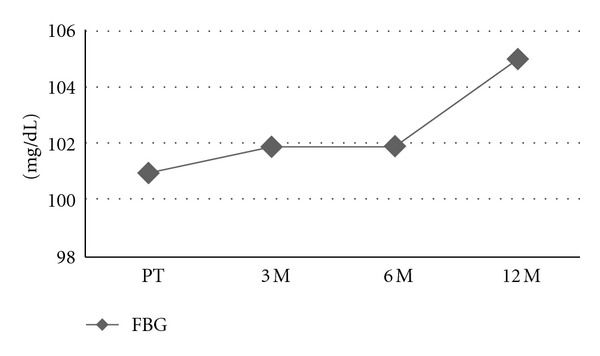
Fasting blood glucose levels during 12-month ADT. FBG: fasting blood glucose, PT: pretreatment, 3 M: 3-months; 6 M: 6 months; 12 M: 12 months.

**Figure 2:**
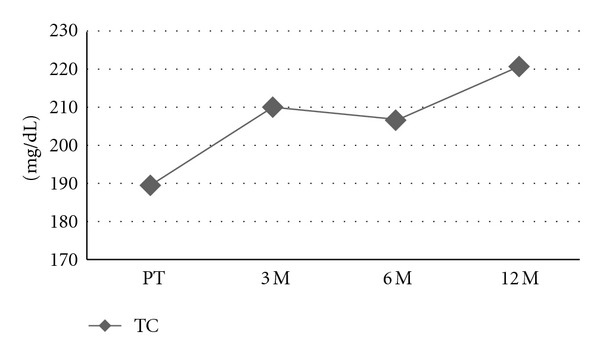
TC changes during ADT. TC: total cholesterol, PT: pretreatment, 3 M: three months; 6 M: six months; 12 M: 12 months.

**Figure 3:**
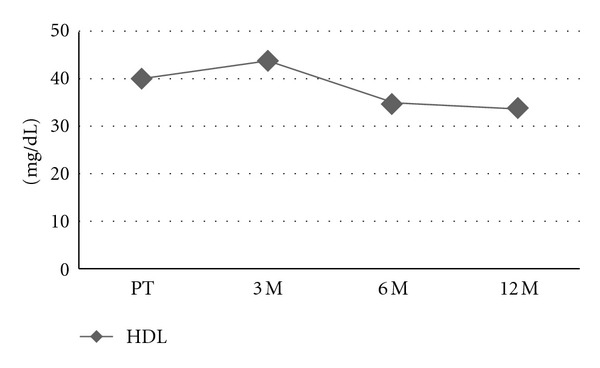
HDL changes during ADT. HDL: high-density lipoprotein, PT: pretreatment, 3 M: 3 months; 6 M: 6 months; 12 M: 12 months.

The mean age of the patients was 74 ± 7 years.  Table 1 shows the basic characteristics of the patients regarding lipid profiles and FBG levels in the pretreatment period and after 3-, 6-, and 12-month treatment periods. FBG, TC, and HDL values during ADT are also presented, in Figures 1, 2, and 3, respectively. After 12 months of initiation, a comparison of GnRH + AA and orchiectomy + AA revealed similar outcomes in terms of FBG, TC, HDL, LDL, and TG (*P* > 0.05).

## 4. Discussion

In this study, we found that the patients receiving ADT had higher TC, LDL cholesterol, and TG levels compared to their own pretreatment levels after three months and through the end of the 12-month initiation of the treatment. HDL cholesterol had a different course during this time; HDL values increased after three months but decreased for the rest of the study period through one year. FBG levels followed a different course; they remained unchanged at six months and increased by 12 months.

In a study conducted on 22 patients, Smith et al. observed that a three-month ADT course with GNRH analog + oral cyproterone acetate did not alter lipid profiles or glucose during treatment [13]. However, Dockery et al. reported in another study, with a small sample size, that ADT increased TC and HDL cholesterol, but not LDL cholesterol or TG, over a period of three months. They used GnRH in all patients except one and prescribed an AA transiently. They also found that fasting glucose levels remained unchanged over the same period, while significant changes in TC and HDL cholesterol were observed within the first three months of treatment; subsequent changes were more modest [14].

In this study, our results were similar to those of Smith et al.; by the end of six months, TC, LDL cholesterol, and TG levels had increased. However, in the study by Smith and colleagues, HDL cholesterol continued to increase over time, while it decreased markedly in our study [13]. Our results showed that FBG levels remained unchanged over this period of time compared to the pretreatment values. Similarly, Nishiyama et al. studied 49 patients with prostate cancer who received ADT in a manner similar to ours, GnRH + AA or orchiectomy + AA, for six months and found a slight, but significant, increase in FBG levels over the course of the study. In the same period, they reported that HDL cholesterol and TG levels did not change significantly [15].

Although they did not provide details regarding ADT, Mohamedali et al. showed that ADT users had significantly higher 12-month FBG levels compared with controls, and they concluded that ADT was the strongest statistically significant, independent predictor of FBS levels [16]. In our study, statistically significant FBG levels were detected one year later. In addition, Basaria et al. studied a limited number of patients, and they observed an increase in FBG levels in patients who received ADT (no detail on ADT) for one year. [17]. In contrast, to assess whether ADT was associated with an increased incidence of diabetes, other cardiac problems, and stroke, Keating et al. extensively studied (mean 4.5 years) a large number of patients (more than 37,000) of all ages who were diagnosed with and treated for prostate cancer. Their patients who received ADT (39% of the total patients) also received treatments that included GnRH antagonists, oral AA therapy, a combination of GnRH antagonists and oral AA therapy, or orchiectomy. They found that after adjusting for patient and tumour characteristics, any use of ADT was associated with diabetes [18]. The aforementioned findings, along with many others suggest that patients with an altered glucose metabolism might require a long amount of time for ADT administration to produce results, as Pagliarulo underlined in a review [19]. 

As Mohamedali stated, studies demonstrating lipid alterations with ADT have been somewhat contradictory [16]. In an early study, chronic administration of the GnRH agonist leuprolide depot was used in 26 BPH patients, and the mean TC level increased by 10.6%, HDL cholesterol by 8.2%, and TG by 26.9%, but LDL cholesterol levels did not change [11]. In our study, serum total cholesterol, LDL cholesterol, and TG levels also increased by the end of one year, but HDL levels decreased. We found that TC increased by 16.3%, LDL cholesterol by 28.3%, and TG by 14.7%, but HDL cholesterol decreased by 9.8% at the end of 12 months. 

Mohamedali et al. reported that ADT users tended to have higher levels of TC, LDL cholesterol, and TG, as well as HDL cholesterol, compared to controls, but none of the differences were statistically significant [16]. Similarly, Smith et al. studied 40 patients with prostate cancer for 48 weeks and found that GnRH agonist treatment increased serum concentrations of TC, HDL cholesterol, LDL cholesterol, and TG by 9.0 ± 2.1%, 11.3 ± 2.6%, 7.3 ± 3.5%, and 26.5 ± 10.0%, respectively, in their participants [20]. In another interim randomised, placebo-controlled study, Smith et al. showed an increase in TC, LDL cholesterol, and TG, and a decrease in HDL cholesterol, after 12-month use of ADT. In this last study, HDL cholesterol decreased, which was in contrast to many others, but agreed with our study's findings [13]. 

The present study had a number of limitations. The first is that this was a retrospective study. The second is the small sample size of the patients who were included in the study. The third limitation is that our patients who received ADT were heterogeneous regarding type of castration; some patients underwent bilateral orchiectomy, while others received GnRH agonists. Our participants who received GnRH could have been divided into three groups: leuprolide 11.25 mg, leuprolide 22.5 mg, and goserelin 10.8 mg. Each of these modalities may be assumed to have a different impact on side effects. In addition, it was not possible for us to make any queries regarding the daily lifestyles and anthropometric examinations of the patients, which could have been important factors to consider in our analysis. 

In conclusion, ADT may cause some alterations in respect to FBG, TC, HDL cholesterol, LDL cholesterol, and TG, depending on the duration of the treatment. A 12-month treatment may increase FBG, TC, LDL cholesterol, and TG, but may decrease HDL cholesterol. These metabolic alterations in addition to prostate cancer may require close followup.

## Figures and Tables

**Table 1:** Pretreatment and one-year treatment values of FBG levels and lipid profiles.

Variables	Pretreatment (mg/dL)	3-month ADT (mg/dL)	6-month ADT (mg/dL)	12-month ADT (mg/dL)	*P* ^*¶*^
FBG_(mean ± SD)_	101 ± 10	102 ± 9	102 ± 9	105 ± 7	0.009
TC_(mean ± SD)_	190 ± 37	210 ± 38	207 ± 39	221 ± 19	0.000
HDL_(mean ± SD)_	40 ± 8	44 ± 8	35 ± 6	34 ± 6	0.000
LDL_(mean ± SD)_	113 ± 32	130 ± 30	137 ± 32	145 ± 12	0.000
TG_(median (min–max))_	129 (55–207)	165 (56–215)	158 (69–199)	148 (88–196)	0.000

FBG: fasting blood glucose, TC: total cholesterol, HDL: high-density lipoprotein, LDL: low-density lipoprotein, TG: triglyceride, ^¶^ *P* values denote the significance of the difference between the pretreatment and 12-month ADT values, SD: standard deviation.
